# Neuronal damage of the dorsal hippocampus induced by long-term right common carotid artery occlusion in rats

**Published:** 2014-03

**Authors:** Wachirayah Thong-asa, Knokwan Tilokskulchai

**Affiliations:** 1 Department of Zoology, Faculty of Science, ASESRU, Kasetsart University, 10900, Bangkok, Thailand; 2 Department of Physiology, Faculty of Medicine Siriraj, Siriraj Hospital, Mahidol University, Bangkok, Thailand

**Keywords:** Delay neuronal death Hippocampal neurons, Mild cerebral hypoperfusion Permanent right common Carotid artery occlusion

## Abstract

***Objective(s):*** The present study investigated the effect of long-term mild cerebral hypoperfusion induced by permanent unilateral (right) common carotid artery occlusion (UCO) on the dorsal hippocampal neurons in rats.

***Materials and Methods:*** Sixty four male Sprague-Dawley rats aged 4 months were divided into two groups of sham and UCO. These two groups were further divided into 4 sets of histopathological observation periods at 8, 16, 48 and 56 weeks after arterial occlusion. Pathological changes were observed in three regions (CA1, CA3 and DG) of the dorsal hippocampus.

***Results:*** Significant increase of damaged neurons in CA1 region at 8, 16, 48, and 56 weeks were observed, whereas in CA3 and DG regions it was at 16, 48, and 56 weeks. Gradual increase of damaged neurons was found without significant change in hemodynamic parameters.

***Conclusion:*** Long-term right common carotid artery occlusion in rats induced delay and progressive damage to the dorsal hippocampus with regional vulnerability from CA1 followed by CA3 and DG regions.

## Introduction

Chronic cerebral hypoperfusion plays an important role in brain dysfunction and damage both in aging and Alzheimer’s disease (1–5). Dysfunction and damage of neurons in the brain as a result of cerebral blood flow (CBF) reduction depend on the location of blood vessel stenosis and duration and degree of CBF reduction (6). Pattern of CBF reduction indicates pattern of neurological disorders. Sudden drop of CBF leads to stroke, resulting in ischemic core and penumbral zone of affected neuron. A moderate but persistent reduction of CBF contributes to the development of dementia and is associated with delayed neuronal damage (7). Causal relationship of CBF reduction, neurodegeneration and cognitive decline was studied using animal model of vessel occlusion. Permanent bilateral common carotid artery occlusion (2VO) in rodents is a popular model. 2VO model clearly revealed short-term and long-term damage both in white and gray matters. Neurodegeneration was indicated since the first week of 2VO and severity progressed with time in correlation with behavioral deficit (8-12). 2VO model could be relevant to the CBF reduction pattern in aging and dementia only 8–12 weeks after the onset of occlusion; however, it was harsh because during this period extensive loss of the hippocampus was observed (7, 13). 

Recently, the permanent unilateral common carotid artery occlusion (UCO) was proposed (14). As a milder cerebral hypoperfusion model, UCO might closely resemble CBF reduction in aging and dementia. Permanent right common carotid artery occlusion indicated only early white matter change at 28 days after the onset of occlusion without hippocampal neurodegeneration. CBF asymmetry was found in the ipsilateral hemisphere after UCO and the ipsilateral CBF returned to baseline within 4 weeks (14). Significant reduction of CBF was not found in long-term occlusion (15). It is interesting that 2VO model indicated a normal CBF within 12 weeks but no correlation with hippocampal neuropathology and behavioral deficits. In UCO model, after the time point which the CBF return to normal is indicated, the pathology in the hippocampus might progress. The present study was interested in long-term effect of mild cerebral hypoperfusion with focus on the dorsal hippocampal neurons which are well known to be vulnerable to global cerebral ischemia.

Therefore, the aim of the present study was to investigate the effect of long-term mild cerebral hypoperfusion caused by permanent right common carotid artery occlusion on the dorsal hippocampal neurons in rats. 

## Materials and Methods

***Animals***

Animal care and experimental procedure were approved by the Animal Ethics Committee, Faculty of Medicine, Siriraj Hospital, Mahidol University, Bangkok, Thailand. Sixty four male Sprague-Dawley rats aged 4 months (450–500 g) from National Laboratory Animal Centre, Mahidol University, Salaya, Nakornprathom were housed in 1 pair per cage at constant room temperature (25^o^C), 12 hr light/dark cycle with free availability of standard diet and filtered tap water. 

***Surgery***

Before surgery, all rats were anesthetized by intramuscular injection of ketamine 40 mg kg^-1^ plus xylazine 5 mg kg^-1^ followed by subcutaneous injection of atropine sulphate 0.05 ml (0.60 mg ml^-1^). After ventral mid-line incision, the right common carotid artery was gently separated from nerves and surrounding tissues then it was occluded permanently with 3-0 silk suture). The same operation without arterial occlusion was performed on control group rats (sham). After surgery, ampicillin (0.125 ml 100 g^-1^) was injected intramuscularly. All rats were allowed to recover from anesthesia under heating lamp and blanket for 30 min or until their wakefulness was seen, then they were returned to their home cages in the housing room. There were thirty-two rats in UCO and thirty-two rats in sham. The physiological parameters such as systolic blood pressure (SBP), diastolic blood pressure (DBP), mean arterial pressure (MAP), and heart rate (HR) were measured in awake and restrained rats using tail cuff method (MAP was calculated using [(2 x diastolic)+systolic] / 3 formula from SBP and DBP data acquired by Chart 5 ADInstruments software). These physiological parameters were measured before surgery (as baseline value), 3 – 8 hr, 24 hr, 8 weeks, 16 weeks, 48 weeks, and 56 weeks after permanent right common carotid artery occlusion. Physiological parameters were interpreted as mean ± SEM. In addition, SBP was interpreted as the difference value from baseline as well. 

***Histopathological observation***

Euthanasia was done via lethal dose injection of ketamine plus xylazine at 8, 16, 48, and 54 weeks after arterial occlusion. Rats were transcardially perfused with 0.9% normal saline solution (NS) for 10 min followed by 4% paraformaldehyde (PFA) in 0.2 M phosphate buffer solution (PBS), pH 7.4 for 10 min. Brain was removed rapidly and post fixed with 4% PFA in 0.2 M PBS. Twenty-four hours later, all brains were processed and prepared for paraffin block. Afterwardsو brain blocks were cut transversely using rotary microtome to 5 µm of thickness. The coronal serial brain sections were collected from Bregma – 2.80 to – 3.80 mm which covered the dorsal hippocampal region (16). Selection of brain sections for histopathological observation by picking up 3 slides (125 µm of space interval) from each rat began from Bregma – 3.14 mm.

***Cresyl violet staining***

Brain sections were deparaffinized in different solutions step by step starting from xylene I, II, 100%, 95%, 80%, and 70% alcohol for 5 min in each step. After that, they were hydrated in distilled H_2_O for 5 min before staining with 1.5% Cresyl violet for 10 min followed by quick washing in 95%, 100% alcohol I, 100% alcohol II, and acetone for 5 min in each step. Finally, brain sections were cleared in xylene and mounted with mounting media. Cresyl violet stained sections were examined under light microscopy (Olympus Tg300). The photomicrograph of the hippocampal regions were captured by separating into three regions including CA1, CA3, and dentate gyrus (DG). From each region in left and right hemispheres, three representative images were captured separately with image size of 493.51 x 290.10 µm under original magnification of 20x. Number of viable and damaged neurons in each cropped image were counted and represented as sum of neurons in three cropped images. Therefore, total observation area of each region was (493.51 x 290.10) x 3 = 0.42 x 10^6^ µm^2^. Numbers of viable and damaged neurons from three sections of each rat were averaged and used for further statistical analysis. Interpretation data were the average number of viable neurons, damaged neurons, and percentage of damaged neurons per area. The latter was calculated using the formula [damaged neurons / (viable neurons + damaged neurons)] x 100.

***Characterization of viable and damaged neurons***

Viable neuron was characterized by properties such as exhibiting a visible nucleus and nucleolus with light purple stain in the cytoplasm. Diameter of cells ranged from 15 to 35 µm in CA1 and CA3 regions and 9 to 25 µm in the DG region. Damaged neuron was characterized by dark purple staining of cresyl violet with cell shrinkage, inability to reveal nucleolus and appearance of vacuole around neuron (Figure 2). Viable and damaged neurons were counted in blind fashion by 2 investigators using ImageTool 3.0. 

**Table 1:** Physiological parameters measured before surgery (baseline), 3 – 8 hr, 24 hr, 8 weeks, 16 weeks, 48 weeks, and 56 weeks after right common carotid occlusion

Time	Groups	Parameters			
		SBP (mmHg)	DBP (mmHg)	MAP (mmHg)	HR (beat/min)
Baseline	Sham	126.08±2.92	113.73±1.51	117.93±1.53	372.18±5.14
	UCO	129.28±1.66	116.13±1.66	120.35±1.36	395.72±6.17
3 – 8 hr	Sham	91.20±7.54*	78.12±2.67 *	82.16±2.83 *	312.95±7.76*
	UCO	77.45±5.09 *	65.68±2.07 *	71.63±2.56 *	304.45±5.54*
24 hr	Sham	127.71±2.80	115.16±2.30	114.75±3.72	379.34±5.22
	UCO	127.11±1.95	127.11±3.47	120.38±1.80	388.43±5.79
8 weeks	Sham	125.02±2.10	113.88±1.66	117.59±1.42	377.47±5.26
	UCO	118.62±5.84	110.21±2.96	112.99±2.99	378.59±7.67
16 weeks	Sham	124.83±1.99	115.18±2.81	117.16±2.75	384.62±11.62
	UCO	122.06±4.07	108.89±2.65	110.70±2.60	393.33±2.72
48 weeks	Sham	127.23±2.00	113.22±1.59	117.90±1.35	410.25±9.37
	UCO	123.39±1.67	110.12±1.28	114.52±2.41	398.57±8.33
56 weeks	Sham	132.77±2.55	112.11±2.57	118.62±2.49	443.66±11.33
	UCO	128.60±3.00	97.20±11.39	105.01±11.49	419.40±13.06

The data was represented as mean ± SEM. Abbreviations: SBP: systolic blood pressure; DBP: diastolic blood pressure; MAP: mean arterial pressure; HR: heart rate; UCO: unilateral (right) common carotid occlusion;

* : *P*-value < 0.05 compared to baseline value

***Statistical analysis***

Viable, damaged and percentage of damaged neurons were analyzed using one-way analysis of variance (ANOVA). A paired *t*-test was used to compare the within group parameters measured before and after surgery. The statistical significance was accepted when *P*-value was less than 0.05.

## Results

***Mortality***

All rats recovered within 24 hr and none died within 16 weeks after occlusion. One rat in UCO group died at 48 weeks and another at 56 weeks after permanent right common carotid artery occlusion. All rats gradually gained their weight throughout the experimental period with no significant changes both within and between experimental groups. 

***Physiological parameters***

**Figure 1:**
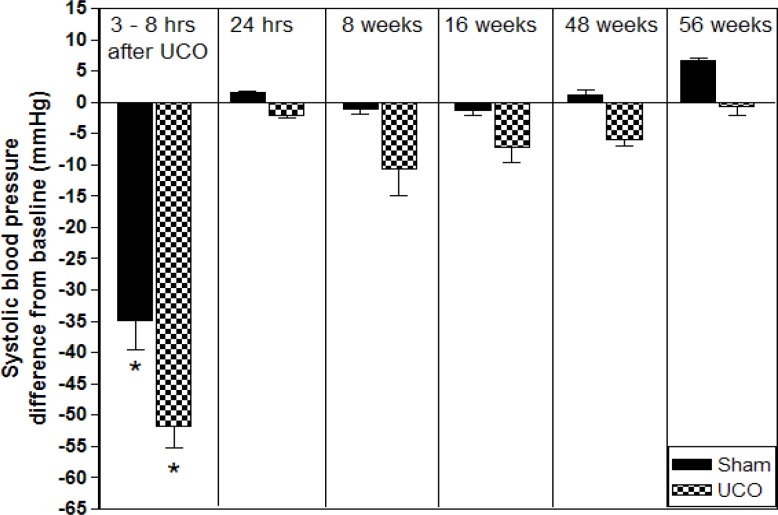
Change in systolic blood pressure (SBP) after right common carotid artery occlusion in rats. SBP was measured in awake and restrained rats using tail cuff method. Abbreviations: Sham: sham-operated control; UCO: unilateral (right) common carotid artery occlusion; hrs: hr; *: *P*-value < 0.05 compared to baseline value

Physiological parameters such as SBP, DBP, MAP, and HR were compared to the baseline value and significant falling was found only 3 – 8 hr after permanent right common carotid artery occlusion (within group). Twenty-four hr later, all physiological parameters returned to normal with no significant differences when compared to baseline value both within and between groups (Table 1 and Figure 1).

***Histopathological observation***

Comparing number of viable and damaged neurons in CA1, CA3, and DG of the dorsal hippocampus between left and right hemispheres indicated no significant difference. Therefore, the data was interpreted as total number of viable and damaged neurons per area in each region of the dorsal hippocampus. 

Viable neurons in CA1, CA3, and DG regions of sham and UCO were progressively reduced. More reduction was found in UCO than in sham. Comparing groups revealed significant differences in viable neurons in CA1 region at 8, 48, and 56 weeks whereas in CA3 region it was at 8 and 16 weeks and in DG region at 16, 48, and 56 weeks, respectively (Figure 3A, B and C). 

On the contrary, progressively increasing damaged neurons were found in CA1, CA3, and DG regions both in sham and UCO. However, more increase was found in UCO than in sham. Comparing groups indicated significant damaged neuron difference in all three regions of the dorsal hippocampus. Significant damaged neuron increase in CA1 region was at 8, 16, 48, and 56 weeks whereas for CA3 and DG regions it was at 16, 48, and 56 weeks (Figure 3 D, E, and F). 

**Figure 2:**
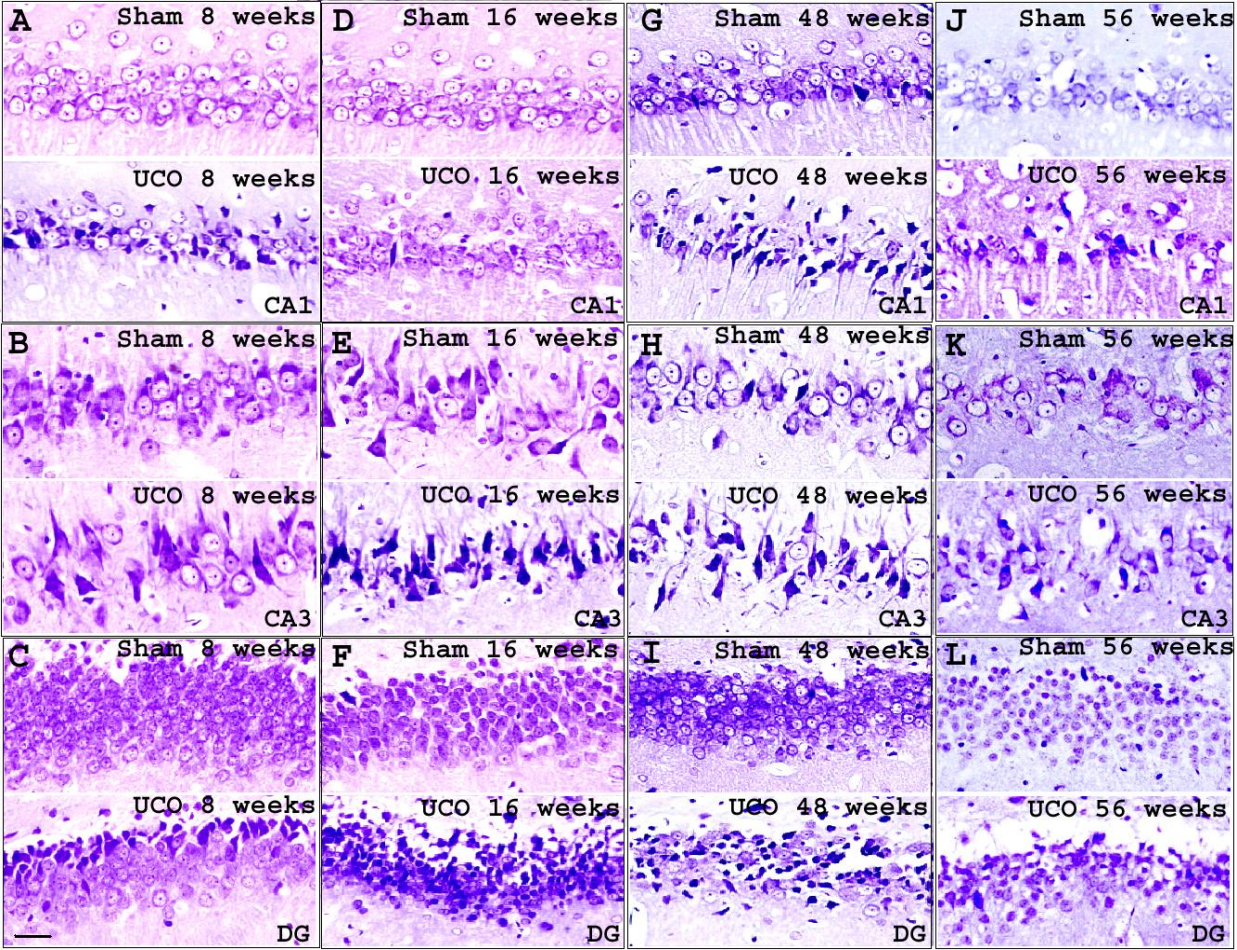
Representative photomicrographs of Cresyl violet staining. Representation of CA1, CA3, and DG regions of the dorsal hippocampus at 8 weeks (A, B, and C), 16 weeks (D, E, and F), 48 weeks (G, H, and I), and 56 weeks (J, K, and L) after right common carotid artery occlusion. Original magnification: 20x; Scale bar: 30 µm

**Figure 3:**
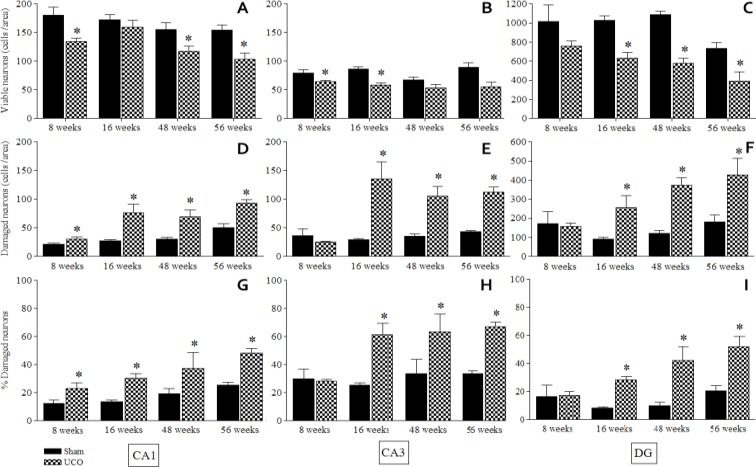
Bar chart of viable, damaged neurons, and the percentage of damaged neurons in CA1 (A, D, G), CA3 (B, E, H), and DG (C, F, I) regions of the dorsal hippocampus at 8, 16, 48, and 56 weeks after permanent right common carotid artery occlusion. Abbreviations: Sham: sham-operated control; UCO: unilateral (right) common carotid artery occlusion; *: *P*-value < 0.05 compared to sham

Comparison of percentage of damaged neurons in each region resembled damaged neuron comparison. Significant increase in percentage of damaged neurons in CA1 region was at 8, 16, 48, and 56 weeks whereas for CA3 and DG regions it was at 16, 48, and 56 weeks (Figure 3 G, H, and I). 

Neurodegeneration of the dorsal hippocampus after long-term right common carotid artery occlusion occurred in delayed fashion and the severity progressed with time. No correlation with hemodynamic parameters was found. 

## Discussion

The present study investigated the long-term role of permanent right common carotid occlusion in inducing delayed neuronal death in the dorsal hippocampus. Damage to the hippocampal neurons was delayed after permanent occlusion of the right common carotid artery. Evidences from previous UCO studies indicate only asymmetrical CBF after permanent occlusion of the right common carotid artery (15). The reduction of CBF was significant only in ipsilateral hemisphere of carotid occlusion. CBF in the ipsilateral hemisphere was suddenly reduced after onset of occlusion then gradually increased and returned to the baseline level within a month without neuronal death in the hippocampus (14, 15). Regrettably CBF was not measured in the present study due to lack of means. However, the CBF pattern in our present study and the previous UCO studies might be identical. Both permanent unilateral and bilateral common carotid artery occlusions indicated that CBF finally returned to baseline (11, 14, 15, 17–19). Most interestingly, normal CBF in 2VO model was not correlated with the progressive neuronal damage in the dorsal hippocampus. Neuronal damage was found in CA1 region from the first week and severity progressed with time. The total hippocampal loss was found at 8 – 13 weeks after bilateral carotid artery occlusion (10, 20, 21). Hippocampal pathology in the 2VO model was harsh and early, unlike our present study, in which it occurred later at 8 weeks after arterial occlusion; however, they were otherwise identical especially during the period of normal CBF. Our present study is the first reporting of the long-term effect of permanent right common carotid artery occlusion on the hippocampal neurons. The results indicated progressively decreased viable neurons in contrast with damaged neurons in the dorsal hippocampus with no difference between left and right hemispheres. Comparison between UCO and sham indicated significant increase of percentage of damaged neurons in CA1 region since 8 weeks after permanent right common carotid artery occlusion, whereas in CA3 and DG regions at was at 16, 48, and 56 weeks later. This result indicated the vulnerability of CA1 hippocampal neurons to global cerebral ischemia, which resembles the previous studies (8, 9, 12, 18). Delayed damage of the hippocampal neurons after permanent right common carotid artery occlusion was probably due to the activation of compensatory mechanisms. Significant hippocampal neuronal damage was not found within 28 days after UCO (14) in addition to delayed fashion of neuronal damage in CA1 region as found in our present study. Compensatory mechanisms such as arterial dilation, angiogenesis, nonperfused capillary recruitment, and activation of biochemical regulation of the CBF (17) which are key roles of nitric oxide and vascular endothelial growth factor (21, 22) might help in restoration of the CBF and prolonging viable neurons after arterial occlusion. Although the compensatory mechanism might help in restoration of the CBF, it might not be sufficient for maintaining the neuronal metabolism in long-term occlusion. Insufficiency of nutrients due to difficulty of transport through the blood brain barrier (BBB) might occur likewise in 2VO model (8, 23). Ultrastructural abnormalities such as basement membrane thickening and fibrous collagen deposits might block nutrient passage through the brain neurons and lead to insufficient ATP sources, decrease in ATPase activity, and increase in lactate concentration as a result of anaerobic Glycolysis as well as significant decrease of N-acetyl aspartate/Creatinine (NAA/Cr) ratio in the hippocampus (24, 25). Therefore, chronic suboptimal metabolism might play an important role in the delayed neuronal death in our present study. Persistent hypoglycemia might lead to disregulation of the neuronal homeostasis. Ischemic damage to the dorsal hippocampal neurons might be associated with change in ionic homeostasis and lead to excitotoxicity. There were two sequences of excitotoxicity; the first was immediate neuronal cell swelling induced by the influx of sodium ions from the outside of the cell, the second was delayd neuronal degeneration induced by excessive calcium influx. Both sequences were involved in N-methyl-D-aspartate (NMDA), 2-amino-3-(3-hydroxyl-5-methylisoxazol-4-yl) proprionate (AMPA) and kinate receptor pathways (26, 27). If the CBF was suddenly dropped as occurred immediately after the onset of arterial occlusion, it would lead to abrupt termination of ATP. In this case, the cell fate was necrotic death which was accompanied by cell swelling and membrane disruption with random DNA breaks, while in moderate CBF reduction (chronic cerebral hypoperfusion), ATP level was persistently low but did not completely cease. In this case, the cell fate was apoptotic death, which was accompanied by condensed chromatin, DNA fragmentation and cell blebbing which appeared as apoptotic bodies. Our present study used only conventional dye which cannot identify the exact type of neuronal death. However, the neuronal death categorization after global cerebral ischemia was not shown as solely a typical necrotic or apoptotic morphology but involved a molecular and biochemical hybrid along the apoptosis-necrosis continuum (28–30). 

Other biological changes were involved in the delayed neuronal damage after ischemic insult, such as reactive microglia which was predominantly in pyramidal cell layer of the hippocampus, where extensive neuronal damage was observed (31). Increasing proinflammatory cytokines (TNF-α, interleukin-1β: IL-1β, interleukin-6: IL-6), were involved in ischemia/hypoxia and trauma-induced brain injury (26, 31). The data from the previous UCO study indicated significant increase of IL-6 at 8 weeks after permanent right common carotid artery occlusion (14). It might be involved in delayed neuronal damage in CA1 area after 8 weeks of UCO; however, its regulation in response to cerebral hypoperfusion remains unclear and long-term observation in UCO model is lacking. 

The present study also measured consequences of permanent right common carotid artery occlusion for hemodynamic parameters. Our results did not resemble those of previous 2VO studies. Permanent bilateral common carotid artery occlusion produced hypertensive response which was indicated by increased blood pressure and heart rate. Measuring the systolic blood pressure using the tail cuff method in unrestrained awake rats from 24 hr to 9 weeks after 2VO indicated significant increased systolic blood pressure over the baseline, which suggested a persistent elevation of the sympathetic tone in 2VO model (7, 11). Our present study found significant decreased systolic blood pressure, diastolic blood pressure, mean arterial pressure, and heart rate during 3 – 8 hr after arterial occlusion. Reduction of these parameters during this period was the result of ketamine plus xylazine anesthesia, which promoted and sustained hypotension in rats  (32). Twenty-four hr after occlusion, none of the hemodynamic parameters were significantly different from baseline. Comparing our present results to the 2VO model revealed the UCO model trend to normal hemodynamic unlike the 2VO model which trends to hypertensive response. 

Recent evident of UCO model related to cognitive abilities (33) reveal that rats with 6 days, 4, 8, and 56 weeks but not 16 weeks of UCO show significant deficit in spatial learning assessed in the radial arm water maze (RAWM) and the Morris water maze (MWM). Related to the neuronal damage found in the present study, only 8 and 56 weeks. Notice that at 16 weeks we found significant damage in CA1 hippocampal neuron, but viable neuron not significant from Sham rat (Figure 3). It might be involve in compensatory mechanisms that was mentioned above (17, 21, 22). 

## Conclusion

Long-term effect of mild cerebral hypoperfusion caused by permanent right common carotid artery occlusion in rats, induced delay and progressive neuronal damage in the dorsal hippocampus with regional vulnerability from CA1 followed by CA3 and DG regions.
